# Is Human Cytomegalovirus Infection Associated with Hypertension? The United States National Health and Nutrition Examination Survey 1999–2002

**DOI:** 10.1371/journal.pone.0039760

**Published:** 2012-07-02

**Authors:** Chao Li, Nithushi R. Samaranayake, Kwok Leung Ong, Hoi Kin Wong, Bernard M. Y. Cheung

**Affiliations:** 1 Department of Medicine, University of Hong Kong, Queen Mary Hospital, Hong Kong, China; 2 Lipid Research Group, Heart Research Institute, Sydney, Australia; FuWai hospital, Chinese Academy of Medical Sciences, China

## Abstract

**Purpose:**

Recent studies have implicated the human cytomegalovirus (HCMV) as a possible pathogen for causing hypertension. We aimed to study the association between HCMV infection and hypertension in the United States National Health and Nutrition Examination Survey (NHANES).

**Methods:**

We analyzed data on 2979 men and 3324 women in the NHANES 1999–2002. We included participants aged 16–49 years who had valid data on HCMV infection and hypertension.

**Results:**

Of the participants, 54.7% had serologic evidence of HCMV infection and 17.5% had hypertension. There were ethnic differences in the prevalence of HCMV infection (P<0.001) and hypertension (P<0.001). The prevalence of both increased with age (P<0.001). Before adjustment, HCMV seropositivity was significantly associated with hypertension in women (OR = 1.63, 95% CI = 1.25–2.13, P = 0.001) but not in men. After adjustment for race/ethnicity, the association between HCMV seropositivity and hypertension in women remained significant (OR = 1.55, 95% CI = 1.20–2.02, P = 0.002). Further adjustment for body mass index, diabetes status and hypercholesterolemia attenuated the association (OR = 1.44, 95% CI = 1.10–1.90, P = 0.010). However, after adjusting for age, the association was no longer significant (OR = 1.24, 95% CI = 0.91–1.67, P = 0.162).

**Conclusions:**

In this nationally representative population-based survey, HCMV seropositivity is associated with hypertension in women in the NHANES population. This association is largely explained by the association of hypertension with age and the increase in past exposure to HCMV with age.

## Introduction

Hypertension, a multi-factorial disease, is a global public health burden. [1], [2] It is a predisposing risk factor for myocardial infarction, revascularization, heart failure, stroke and renal failure. Both genetic and environmental factors are involved in the development of hypertension. There are many known environmental factors leading to hypertension. Obesity, inappropriate diet, stress, lack of physical activity, tobacco smoking, alcoholic drinking and certain medications are known risk factors for hypertension. [3] In recent years, meta-analysis of genome wide association studies has highlighted a number of genetic variants associated with hypertension. [4].

Human cytomegalovirus (HCMV), a member of the beta herpes virus family, is a ubiquitous pathogen that infects only humans. [5] It is an opportunistic infection in 90% of the HIV carriers and a latent infection in over 50% of the general population. [6] Although HCMV infection is usually asymptomatic in healthy adults, it can infect organs and tissues virtually throughout the body. HCMV is a cause of congenital diseases, causes primary infection in children and young adults, and has been implicated in pneumonitis, retinitis and gastroenteritis in adults, particularly in immunosuppressed or immunocompromised patients, such as transplant recipients and patients with HIV infection. [7].

Recent studies have suggested that HCMV infection is associated with a significantly increased risk for all-cause and cardiovascular diseases (CVDs) related mortality. [8] It has been implicated in endothelial dysfunction [9] and different cardiovascular diseases, such as myocarditis, atherosclerosis and coronary artery disease. [10], [11], [12] However the association between HCMV infection and hypertension is still unclear. [13] As hypertension is an important cause of CVDs, we studied the association between HCMV and hypertension in the United States (US) National Health and Nutrition Examination Survey (NHANES) 1999–2002.

## Methods

### Study Population

For this analysis, data were extracted from NHANES 1999–2002, which was a continuous survey since 1999. The study protocol was approved by the Centers for Disease Control and Prevention Institutional Review Board. All participants gave written consent for their information to be stored in the database and used for research. They also gave written consent for their blood and urine samples to be kept for future research, such as the measurement of HCMV specific IgG. For participants aged 16–17 years, written consent was obtained both from them and their parents or guardians. Participants were examined in well-equipped mobile examination centers located around the US. The detailed measurement procedures and protocols have been described in previous publications and also on its website. [14] Participants were selected using a stratified, multistage probability sampling design from the civilian non-institutionalized US population. Among the 6610 participant aged ≥16 years who had valid data on HCMV infection and hypertension, 6303 participants with valid data on body mass index (BMI), blood pressure, diabetes and hypercholesterolemia were included in our study.

### Variables of Interest

Race/ethnicity was based on self-reported questionnaires. Participants were classified as non-Hispanic whites, non-Hispanic blacks, Mexican Americans and others. HCMV infection status was determined by HCMV specific IgG test using commercial enzyme linked immunosorbent assay (ELISA) kits (Quest International Inc, Miami, FL) in a centralized manner. Sera with values near the ELISA cutoff (approximately 5.2% of total) were measured again with another commercial ELISA kit (bioMerieux Inc, Durham, NC) to confirm the results. An immunofluorescence assay (Bion International Inc, Park Ridge, IL), was used to give the final results if the results from the first two tests disagreed (approximately 2.7% of total). The optical density from the ELISA assay was reported as an approximate measure of antibody titer. A low and positive optical density reflects a low antibody titer and a high optical density reflects a high antibody titer. BMI was calculated as the weight in kilograms divided by the square of the height in meters. Blood pressure was measured manually by a trained operator using a mercury sphygmomanometer. Hypertension was defined as self-report of diagnosed hypertension, blood pressure ≥140/90 mm Hg, or use of anti-hypertensive medications. Diabetes was defined as a fasting glucose of ≥7.0 mmol/L (126 mg/dl), non-fasting glucose of ≥11.1 mmol/L (200 mg/dl), previous diagnosis by a doctor, or use of anti-diabetic medications. Hypercholesterolemia was defined as total serum cholesterol ≥240 mg/dL, previous diagnosis by a doctor, or taking prescribed medicine for hypercholesterolemia.

### Statistical Analysis

Data were analyzed using the complex sampling function of SPSS version 20.0 (IBM Corporation, Armonk, NY). The four-year mobile examination weight was used in all analyses to account for non-response bias, and the oversampling of blacks, Mexican Americans and the elderly in NHANES 1999–2002 so that the estimates were nationally representative according to the 2000 US Census population. P values were obtained using logistic or multiple regression analysis where appropriate, using the complex sampling function. Logistic and multiple regressions were used to adjust for covariates. Variables that significantly differed between participants with and without HCMV infection, or with and without hypertension, were used as covariates in the regression models. There was no significant interaction between HCMV seropositivity and race/ethnicity with hypertension in multiple regression analysis. Therefore all the racial/ethnic groups were combined for analysis.

## Results

Among the 6303 participants aged 16–49 years in NHANES 1999–2002, 3975 had HCMV infection (54.7±1.3%) and 939 had hypertension (17.5±0.8%). All the percentages were calculated after weighting to the 2000 US Census population.

Table 1 shows the characteristics of all participants according to HCMV seropositivity status. Participants with HCMV infection were older (P<0.001) and more likely to be women (P<0.001), compared to participants without HCMV infection. There were significant racial/ethnic differences between participants with and without HCMV infection (P<0.001).

**Table 1:** Clinical characteristics of all 6303 subjects among subjects with and without serologic evidence of HCMV infection.

Characteristics	HCMVinfection	No HCMVinfection	P
n	3975	2328	
Age, years	34.0(0.3)	32.1(0.3)	<0.001
Men, %	44.3(1.2)	56.1(1.0)	<0.001
BMI, kg/m^2^	27.5(0.2)	27.1(0.2)	0.156
Race/ethnicity, %			
Non-Hispanic white	52.8(2.2)	82.6(1.3)	<0.001
Non-Hispanic black	16.2(1.9)	5.9(0.7)	
Mexican American	14.3(1.5)	3.5(0.6)	
Others	16.7(2.4)	8.0(1.2)	
Diabetes, %	3.6(0.4)	2.8(0.5)	0.189
Hypercholesterolemia, %	21.5(0.7)	20.6(1.0)	0.456
Hypertension, %	18.3(1.0)	16.4(1.2)	0.179
SBP, mmHg*	115.6(0.5)	115.9(0.5)	0.575
DBP, mmHg*	71.8(0.4)	71.5(0.4)	0.563

Data are expressed as mean or percent (SE). BMI, body mass index. SBP, systolic blood pressure. DBP, diastolic blood pressure.

* 241 subjects taking anti-hypertensive medication were excluded from the analysis (n = 165 for HMCV infection group and n = 76 for no HCMV infection group).

Table 2 shows the characteristics of all participants according to hypertension status. Participants with hypertension were older (P<0.001) and had a greater BMI (P<0.001), compared to participants without hypertension. Also hypertensive participants were more likely to be men (P = 0.004), and have diabetes (P<0.001) as well as hypercholesterolemia (P<0.001). There were significant racial/ethnic differences between participants with and without hypertension (P<0.001).

**Table 2:** Clinical characteristics of all 6303 subjects among subjects with and without hypertension.

Characteristics	Hypertension	No Hypertension	P
n	939	5364	
Age, years	38.0(0.5)	32.1(0.3)	<0.001
Men, %	56.4(2.5)	48.2(0.9)	0.004
BMI, kg/m^2^	31.5(0.3)	26.4(0.1)	<0.001
Race/ethnicity, %			
Non-Hispanic white	64.2(2.1)	66.8(1.9)	<0.001
Non-Hispanic black	17.3(2.2)	10.3(1.2)	
Mexican American	7.1(1.1)	9.9(1.1)	
Others	11.3(1.9)	13.1(1.9)	
Diabetes, %	8.6(1.2)	2.1(0.3)	<0.001
Hypercholesterolemia, %	40.7(2.4)	16.9(0.6)	<0.001
HCMV, %	57.4(2.3)	54.1(1.4)	0.179
SBP, mmHg*	129.9(0.9)	112.7(0.3)	<0.001
DBP, mmHg*	81.4(0.7)	69.6(0.2)	<0.001

Data are expressed as mean or percent (SE). BMI, body mass index. SBP, systolic blood pressure. DBP, diastolic blood pressure.

* 241 subjects taking anti-hypertensive medication were excluded from the analysis (n = 165 for HMCV infection group and n = 76 for no HCMV infection group).

**Figure 1:**
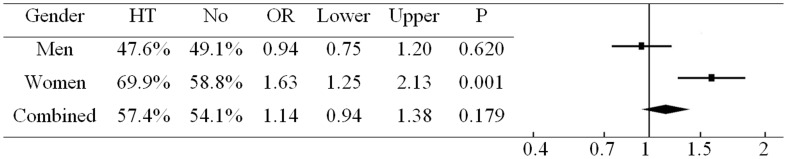
Percentage of HCMV seropositivity in participants with and without hypertension before adjustment. HT, hypertension.

Figure 1 shows univariate logistic regression analysis of hypertension. In this analysis, HCMV seropositivity was associated with hypertension in women (OR = 1.63, 95% CI = 1.25–2.13, P = 0.001) but the association in men was not significant (OR = 0.94, 95% CI = 0.75–1.20, P = 0.620).

Table 3 shows the logistic regression analysis after adjusting for covariates. There was no significant association between HCMV seropositivity and hypertension in men in any of the models. The association between HCMV seropositivity and hypertension in women remained significant after adjusting for race/ethnicity (OR = 1.55, 95% CI = 1.20–2.02, P = 0.002). Further adjustment for BMI, diabetes status and hypercholesterolemia attenuated the association (OR = 1.44, 95% CI = 1.10–1.90, P = 0.010). However, after adjusting for age, the association was no longer significant (OR = 1.24, 95% CI = 0.91–1.67, P = 0.162).

Table 4 shows the association between optical density for the HCMV specific IgG assay and blood pressure as a continuous variable among participants who were not taking any antihypertensive medication. In men, there was no significant relationship between optical density for the HCMV specific IgG assay and systolic or diastolic blood pressure before adjustment (P = 0.144). After adjusting for race/ethnicity, there is a significant association between optical density for the HCMV specific IgG assay and diastolic blood pressure (P = 0.033). Further adjustment for body mass index, diabetes status and hypercholesterolemia did not attenuate the association (P = 0.029). However, after adjusting for age, the association was no longer significant (P = 0.815). In contrast, optical density for the HCMV specific IgG assay was associated with both systolic and diastolic blood pressure in women, but this association was no longer significant after controlling for age. The conclusions remained the same if we included participants on antihypertensive medication but added 10/5 mmHg to their blood pressure on treatment. [15].

**Table 3:** Association of HCMV seropositivity with hypertension in men and women.

	Model 1	Model 2	Model 3
Men			
OR	0.96	1.00	0.93
95%CI	0.75–1.23	0.77–1.30	0.71–1.21
Women			
OR	1.55	1.44	1.24
95%CI	1.20–2.02	1.10–1.90	0.91–1.67
P	0.002	0.010	0.162

Model 1: adjusted for race/ethnicity (non-Hispanic white, non-Hispanic black, Mexican Americans, and others).

Model 2: further adjusted for BMI, diabetes and hypercholesterolemia.

Model 3: further adjusted for age.

## Discussion

Our study used data from the large and nationally representative NHANES study to investigate the association between HCMV and hypertension. It shows that there is no strong evidence to support HCMV as a significant cause of hypertension. Although HCMV seropositivity was significantly associated with hypertension in women, the association was greatly diminished after adjusting for age. This means that the association of hypertension with HCMV seropositivity in women can largely be explained by the increased likelihood of having had HCMV infection with age. A similar trend also appeared in the association between HCMV seropositivity and diastolic blood pressure in men. A previous study involving 1074 women and 857 men (aged 24–39 years) from the Cardiovascular Risk in Young Finns study has shown that CMV antibody titers were independent determinants for systolic and diastolic blood pressure elevation and flow-mediated dilation in men, but not in women. Discrepancies between the Cardiovascular Risk in Young Finns Study and our study might be due to differences in sample size, age range, socioeconomic factors, dietary habits and climate. [16] However, our study has the advantages of having a larger sample size, wider age range and several racial/ethnic groups.

**Table 4:** Association of optical density for the HCMV specific IgG assay with blood pressure in men and women.

	Systolic Blood Pressure			Diastolic Blood Pressure	
	B	P		B	P
Men (N = 2874)					
Unadjusted	−0.133		0.635	0.425	0.144
Model 1	−0.327		0.249	0.661	0.033
Model 2	−0.291		0.309	0.643	0.029
Model 3	−0.449		0.095	0.067	0.815
Women (N = 3188)					
Unadjusted	0.794		0.006	0.638	0.003
Model 1	0.681		0.019	0.772	0.001
Model 2	0.393		0.137	0.630	0.004
Model 3	−0.181		0.484	0.050	0.813

Model 1: adjusted for race/ethnicity (non-Hispanic white, non-Hispanic black, Mexican Americans, and others).

Model 2: further adjusted for BMI, diabetes and hypercholesterolemia.

Model 3: further adjusted for age.

Although no strong evidence that HCMV is a significant cause of hypertension has been found in clinical studies, there is a theoretical basis for this association and several mechanisms can be envisaged. First, HCMV infection could lead to oxidative stress and endothelial dysfunction. [9], [11], [17], [18], [19], [20] As endothelial dysfunction and inflammation are two key mechanisms in the development and progression of hypertension, [21], [22] this provides an explanation of how chronic HCMV infection may result in hypertension. Second, HCMV infection triggers the proliferation of one type of T lymphocytes, CD8+ cytotoxic T lymphocytes. [23], [24] The clonal expansion of HCMV-specific T lymphocytes may impair the response to other antigens. It is also known that T lymphocytes take part in the alteration of vascular tone encountered hypertension induced by angiotensin II, which is a potent vasoconstrictor. [25] Therefore, T cells may be a link between HCMV infection and hypertension. Third, recent research has suggested that microRNAs (miRNAs), a class of post-transcriptional gene expression regulators, are present in the circulation and may serve as potential biomarkers. [26] From a genetic point of view, circulating miRNAs carry disease-specific information owing to inherent genomic alterations. A recent study showed HCMV infection was significantly correlated with the miRNA (hcmv-miRUL112) which is encoded by HCMV and highly expressed in hypertensive patients compared to healthy control subjects. [27] This suggested a possible link between HCMV infection and hypertension at the genomic level as well. Fourth, mouse cytomegalovirus (MCMV), the animal counterpart of HCMV, has been demonstrated to activate over-expression of angiotensin II in the renin-angiotensin-aldosterone system (RAAS), which plays an important part in the emergence of hypertension. [28] Angiotensin II causes vasoconstriction, and also stimulates generation and secretion of ROS, inflammatory cytokines, chemokines and adhesion molecules. [29] Therefore it is possible that HCMV has similar effects on the human RAAS, so further research to elucidate the role of HCMV in hypertension is still needed.

This study has provided some important insights into HCMV and hypertension. The results derived from a large nationally-representative population are reliable due to good sampling design and quality control. It shows that age may account for a large part of the association of HCMV infection with hypertension. Some risk factors known to be closely related to hypertension, such as alcohol consumption, smoking and physical activity, contribute to the development and progression of hypertension. In this study, we did not include these factors as covariates in the adjustment of the association of HCMV with hypertension because there were a substantial number of subjects with missing values in these factors in the NHANES database. Incorporating these risk factors in our model would have reduced the sample size and thereby the power of our analysis. Further studies with larger sample sizes and more comprehensive information on risk factors are needed to elucidate the complex relationship between HCMV and hypertension.

In conclusion, HCMV seropositivity is associated with hypertension in women in the NHANES population. This association is largely explained by the association of hypertension with age and the increase in past exposure to HCMV with age.
